# Whole-genome sequence and annotation of *Salmonella enterica* subsp. *enterica* strain QazSL-4

**DOI:** 10.1128/mra.00876-23

**Published:** 2024-04-16

**Authors:** Sabyrkhan M. Barmak, Ian H. Mendenhall, Yuriy A. Sinyavskiy, Aidar B. Berdygaliev, Turegeldy Sh. Sharmanov, Gulmira T. Sultankulova, Dilyar N. Tuigunov, Elena V. Zholdybayeva

**Affiliations:** 1 Department of Biology and Biotechnology, Al-Farabi Kazakh National University, Almaty, Kazakhstan; 2 Laboratory of Food Biotechnologies and Specialized foodstuffs, Kazakh Academy of Nutrition LLP, Almaty, Kazakhstan; 3 Programme in Emerging Infectious Diseases, Duke-NUS Medical School, Singapore, Singapore; 4 Department of Pediatric Surgery, Asfendiyarov Kazakh National Medical University, Almaty, Kazakhstan; 5 National scientific shared laboratory of biotechnology, National Center for Biotechnology, Astana, Kazakhstan; University of Maryland School of Medicine, Baltimore, Maryland, USA

**Keywords:** whole-genome sequence, annotation, *Salmonella*

## Abstract

Here, we present the whole-genome sequence of *Salmonella enterica* subsp. *enterica* strain QazSL-4 isolated from a chicken fillet in 2018, Almaty, Kazakhstan. The genome obtained using Illumina MiSeq technology consists of 49 contigs with a total length of 4,711,816 bp with a GC content of 52.1%.

## ANNOUNCEMENT

We present here the whole-genome sequence of the Kazakhstan strain QazSL-4 serovar *Salmonella enterica* subsp. *enterica*. The bacterium was isolated from a sample of chicken fillets, randomly selected in Almaty’s retail market in 2018 as a part of monitoring studies of food products. *Salmonella* was isolated using Endo and Levin differential diagnostic media. Isolation and identification of *Salmonella* were carried out by standard methods described in regulatory documents (1). The bacterium *Salmonella* was sown in buffered peptone water, depending on the type of sample at 37°C for 24 hours, followed by selective enrichment according to Rappaport-Vassiliadis) and tetrathionate broth at 42°C and 37°C for 24 hours. *Salmonella* serotyping was carried out using conventional methods of agglutination and PCR (2). The following specific primers for the identification *Salmonella* were used in this work: SE-F: AGGTGACGCTATTGCCGGCAT; SE-R: ATGCGGGGATCTGGGCGA; and SE-probe: FAM-ATTTCGGTGGGGATGACTCGCCAT-BHQ-1.

Genomic DNA isolation was performed using the PrepMan Ultra Sample Preparation Reagent Kit (Thermo Fisher Scientific Inc., Waltham, MA, USA). Then, 150 ng of the total genomic DNA from each isolate of QazSL-4 *Salmonella enterica* subsp. was used for sequencing. Library preparation and Illumina MiSeq sequencing were performed using the Nextera DNA Flex Library Prep Kit and a MiSeq Reagent Kit v3 with 300 bp paired-end reads (600 cycles) according to the manufacturer’s instructions. Quality assessment of the sequencing data (in FASTQ format) was done using FastQC v0.11.15 (3), followed by trimming of adapters and low-quality bases with a Phred quality score of less than 20 using Trimmomatic v0.39 (4). Genomes were assembled using the SPAdes assembler v3.13.2 (5) using a k-mer length of 127 with the “-careful” mode. The resulting assembly had a consensus length of 4,711,816 bp spanning 49 contigs, with an *N*_50_ value of 491,954 bp and an *L*_50_ value of three contigs. The number of reads in total—3,685,536 reads. The genome sequencing project was annotated using the NCBI Prokaryotic Genome Annotation Pipeline (6). Genome annotation predicted 4,521 coding sequences, 8 rRNA operons, 78 tRNAs, 15 ncRNAs, and 2 CRISPR arrays.

The genome project of *Salmonella enterica* subsp. *enterica* strain QazSL-4 was tested for antibiotic resistance genes using the ResFinder 4.1 comprehensive antibiotic resistance gene database (7–9), which predicted the presence of a single resistance gene homolog aac(6′)- Iaa.

The genome sequence was analyzed for prophage content using the PHAge Search Tool Enhanced Release server (10, 11). Analysis by the PHASTER program revealed 10 prophage regions, two intact regions, and eight incomplete regions.

Using SPIFinder 2.0 (12), the presence of widespread *Salmonella* pathogenicity islands of SPI-1, SPI-2, SPI-3, SPI-4, SPI-5, SPI-9, SPI-10, SPI-13, and SPI-14 was detected.

## Data Availability

A genome-wide sequence project for *Salmonella enterica* subsp. *enterica* strain QazSL-4 has been deposited in the NCBI GenBank database under the accession number NZ_JAJHZK000000000. The associated BioProject, Assembly, and BioSample accession numbers are PRJNA770501, GCF_020861915.1, SAMN22222384, and SRR25711269, respectively.
